# Friends in need: The value of supportive colleagues in research

**DOI:** 10.1002/hem3.70223

**Published:** 2025-09-22

**Authors:** Freda K. Stevenson, Federico Caligaris Cappio

**Affiliations:** ^1^ School of Cancer Sciences, Faculty of Medicine University of Southampton UK; ^2^ AIRC (the Italian Association for Cancer Research) Milano Italy

Sometimes the way research in science and medicine works seems far out of the public reach. Yet we know that it is carried out by ordinary people who happen to have followed a path which interests them, and which might carry the benefit of helping patients. As for most human activities, it can be a difficult world racked with ambition, competition, emotion, and deception. It can also be fascinating and rewarding. The historical career structure was made for men and it has always been difficult for women. In the end, the zigzag path to achievement depends on determination, talent, and on luck. One aspect not often mentioned is the importance of friendship between colleagues, often soured by competition. Our story is of a platonic partnership, in research into chronic lymphocytic leukemia (CLL), between two individuals, one an Italian male clinical scientist and the other a British female scientist (Figure 1). We did not work directly together, but in parallel, sharing the good things and supporting each other through the bad. The results of our efforts were to help to transform our knowledge of CLL, with insight into the biology providing the best prognostic indicator for patients and clinicians. The expansion of understanding then formed a foundation for novel targeted therapies. Our careers in translational hematology reflect the twists and turns of research during our time. Things have changed, but the excitement and fun of research remain for the next generations to enjoy.

**Figure 1:**
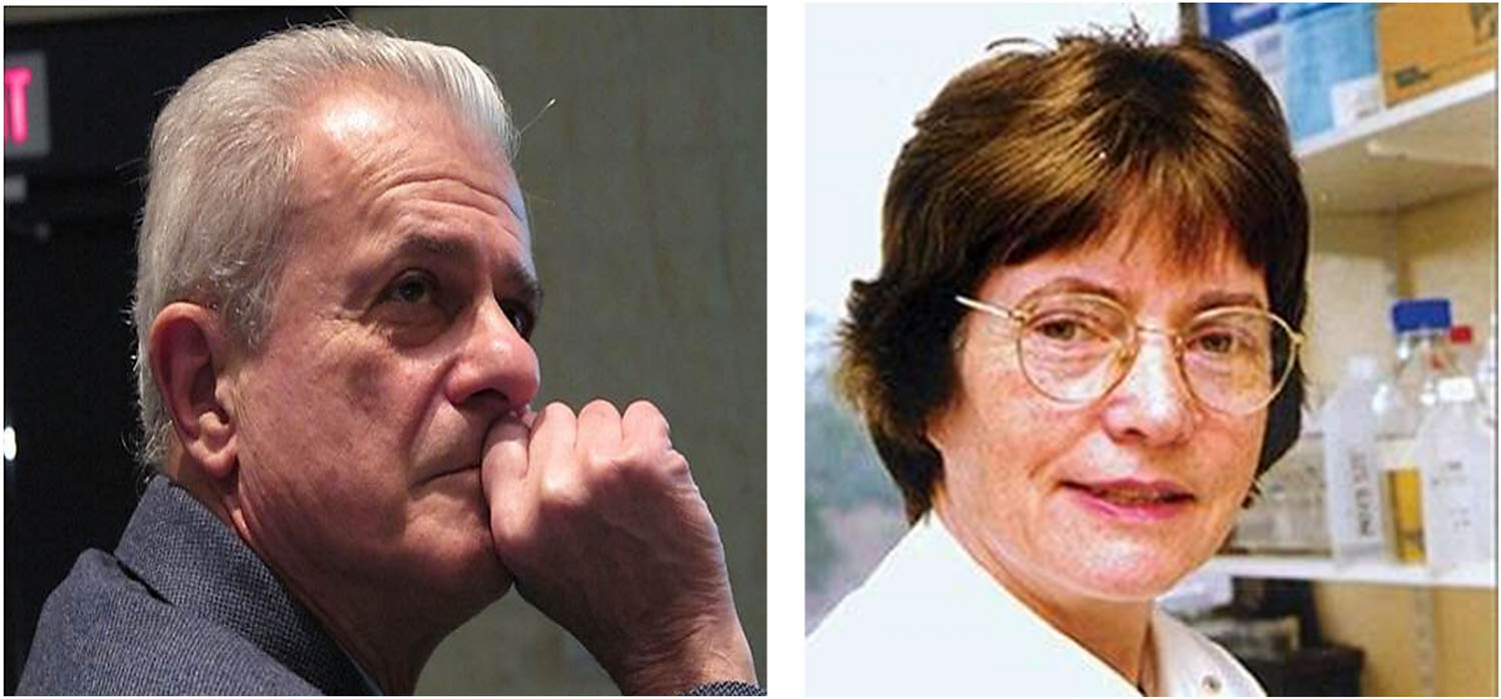
**Federico in a thoughtful mood and Freda in her beloved laboratory**.

## PROBLEMS IN GETTING ON THE LADDER

*Freda:* In my time it was difficult for a lone woman to make a difference in medical research. Not only did society load on all the expectations which go with being female, especially when there are children, but the male‐dominated structures blocked progress. I look back at a career in what became “translational science,” operating at the interface between the laboratory and the clinic, beginning in the 1960s to the present. Many things have improved, with more awareness by institutions and male colleagues of problems facing women, but there are still many challenges. Hematology has always been open to science, but the power structure lies with clinicians, so results from the laboratory have to be first, comprehensible, and second, relevant to human disease.

It might seem odd now but, although I went through the usual undergraduate/postgraduate training, emerging with a DPhil from Oxford, I gave little thought to my career. The overwhelming ambition for women was to marry and have children so that is what I did. But, since my husband, George, was Australian and we moved there, I cheekily applied for a Lectureship in Biochemistry at Sydney University. In those days there was a shortage of applications and I forgot to mention that I was pregnant so I became a lecturer. This was pivotal because I was thrust into the academic structure and forced to find child care. It is obvious now that this is a way to go but I was a sort of pioneer, with no help available. On returning to Oxford in 1970, and then moving to Southampton in 1973, I decided to continue this route. By this time a creeping ambition to pursue a research career in cell biology had solidified and I continued, now with three children. Two major obstacles emerged: first, Southampton University was then unknown for basic haematological research, which meant no support due to reputational excellence; second, because I initially worked with George (not for long), it was assumed that all the ideas came from him. However, these disadvantages were counterbalanced by strong financial support from the Tenovus charity based in Cardiff which had endowed the Southampton laboratory and was receptive to grant applications. In the current darker days, it reminds us that investment is essential to build a team and to deliver novel findings. Even so it took years of publications and conference participation to establish my own reputation and this is where the support from Federico and others mattered.

*Federico:* Italian science had different but parallel problems. I was born in a small mountain village so in a sense I came from nowhere. I graduated in Medicine in 1973 at the University of Torino. Those days, to pursue an academic career, one had to comply with three rules almost etched in stone: to be male, exempt from military service and belong to a wealthy family. Except for the male sex this was not my situation and, after having served in the army, I had to accept extra jobs such as night shifts to earn a living while working daily in a university clinical department. The relationship with patients was a most enriching experience that triggered a passion for investigating how to overcome the biological barriers that protect tumours, and how to find ways to improve diagnosis and treatment.

In 1981, I had a most instructive post‐doc experience in London at the Department of Immunology, Royal Free Hospital under the guidance of George Janossy. I was fascinated by the mechanistic approach to patient's investigation as opposed to descriptive observational studies. I decided to take this strategy to Torino and apply it to CLL. This made me enter the international conference circuit where I met Freda who had a key role in persuading me that immunology was reshaping clinical disciplines, namely hematology. International connections proved essential to have the support of my boss within the cabals of influential men. I was appointed Professor of Medicine in 1990. In 2003, I moved to San Raffaele University, Milano, where I founded the Departments of Oncology, of Onco‐Hematology, and the Research Division of Molecular Oncology. I asked Freda to be my advisor and she was key to the scientific success of the program project on CLL and multiple myeloma funded by the charity *Associazione Italiana per la Ricerca sul Cancro (AIRC)* highlighting the central role of the microenvironment in B‐cell tumors.

## ACCEPTANCE OF NEW IDEAS

For Freda, the first focus of research was on DNA vaccines against lymphoma. An important lesson was learned: even though she developed an effective fusion vaccine, and thought clinical trials would follow naturally, it soon became clear that there was no interest in vaccination from the pharmaceutical companies, who were (it turns out wrongly) negative about “genetic vaccines.” If COVID had any positive influence, it was to change this view, and her mentees in Southampton are now running clinical trials in Liverpool of a similar design against lung cancer.

Facing the brick wall, she had to develop a new direction. Another lesson was that progress depends on emerging technology, and she embraced the new science of immunogenetics. Applying this to B‐cell tumors was obvious and she looked at CLL, previously a “boring accumulation of small lymphocytes.” Federico was applying his newly acquired experience in monoclonal antibodies to reveal the unusual phenotype of CLL cells.^1^ Freda looked at the immunogenetics of the B‐cell receptor, which turned out to be a gold mine, dividing the disease into two separate groups derived from pre‐germinal centre (GC) or post‐GC cells, with important differences in prognosis revealed from the matched clinical data of Terry Hamblin and Nicholas (Nick) Chiorazzi.^2^, ^3^ The history of the discovery of the importance of the two subsets of CLL was subsequently published by Nick and Freda to celebrate the 20th anniversary in *HemaSphere* in 2020.^4^

The European Hematology Association (EHA) saw the importance and both were invited speakers at conferences organized by the EHA to share information on the exciting possibilities for the focused treatment of CLL. In 2014, Freda received the *Jean Bernard Lifetime Achievement Award* from EHA for her contributions to the advancement of hematology. Although she received subsequent awards, this was a precious first recognition of her findings. Freda and Federico combined their knowledge in a review of the phenotype and genotype of CLL.^5^ They also shared a project on the nature of anergy in CLL, with one of Federico's talented research fellows, Benedetta Apollonia, spending time in the Southampton laboratory.^6^ Importantly, the B‐cell receptor turned out to be a target for inhibitory drugs, with new and effective therapies for the more aggressive subset showering into the clinic.

## MUTUALITY

Support from male colleagues was rare in a competitive world, and the partnership of Freda with Federico was fortunate. Perhaps Federico not being from the United Kingdom, where the old‐boy network often worked against females, helped. Although we knew each other for some years, we became much closer at a relatively late stage of our careers when we were both in our 50s. Three circumstances had a major influence in cementing our friendship and mutual respect. These illustrate some of the challenges and joys of lives in research.

### Iran 1996

Freda was asked by Iranian researchers to lecture at a meeting in Shiraz and she was told she could bring a European medical scientist with her. Although she hardly knew Federico at that time, he seemed appropriate for the task and accepted her invitation to join. One joy of research is that sharing findings at conferences allows insight into different societies. It was a real privilege to meet young Iranian students and to be taken to see the historical sites at Persepolis and Isfahan. It was a little offset by being stopped by the armed police and being told to tell Freda to cover her hair more completely, and by the lack of wine! The meeting “Shiraz University Oncology Meeting May 1996” was an important event for the Iranian scientific community held in the University amphitheater full of silent students. On one side serious males, on the other separated side the black wall of females all covered with chador and veil. The introduction was a long loud prayer from the mullah, questioning the value of medical research. By good fortune, Freda's first slide was of Edward Jenner's vaccination against smallpox, and this loosened the audience. Two English‐speaking male students escorted us everywhere as “bodyguards,” interested in western research organization, cautious when asked about their country, and shyly asking about the possibility of coming west to do research. Such academic connections, which operate across political barriers, must surely be of real value in an increasingly fractured world.

### The International Workshop on Chronic Lymphocytic Leukemia (iwCLL)

The iwCLL was established in 1979 by two prominent clinicians in the field, Jacques Louis Binet and Kanti R. Rai. It holds a biennial scientific congress whose highlight is the Rai Binet Medal, which recognizes outstanding contributions to CLL research. At the X iwCLL meeting organized by Federico in Stresa, Italy, the 2003 Medal was awarded to Nick Chiorazzi (USA) and Terry J Hamblin (UK) for “*their discovery of the importance of VH mutations*” published in two back‐to‐back *Blood* papers.^2^, ^3^ In the UK, it was Freda who had initiated the idea of investigating the pathogenetic and prognostic significance of *IGHV* gene mutational status in CLL. She proposed the division of CLL into two distinct subsets based on the cell of origin, and was the senior author of the UK paper. The fact that the all‐male committee failed to recognize her contribution cut deep.

This disharmony was somehow amended in 2015 at the XVI iwCLL meeting, Sydney, Australia. Justice finally prevailed with the announcement that one of the 2015 Rai Binet Medal awardees was Freda K. Stevenson for “identifying the correlation of CLL IGVH with stages of normal B lymphocyte maturation and the correlation of IGVH mutational status as a robust prognostic marker in CLL.” An unexpected and pleasant surprise was a co‐award to Federico Caligaris‐Cappio for “his work on CD5+ B cells, speculating that these were precursors of CLL, and identifying the phenotype, genotype, and function of anergic B cell clones in CLL*.*” It was such a pleasure to share the award, especially in the wonderful surroundings of Sydney.

### AIRC: Giving back

Federico left academic research on January 1, 2016 to become the Scientific Director of *AIRC*, the foremost charity that supports cancer research in Italy, with grants and fellowships evaluated by international peer review. He decided to deal with science policy because, having had a rewarding life in scientific research, he deemed it important to return something, especially to the younger generations. He implemented an AIRC call fostering the independence of junior scientists returning to Italy after a successful experience abroad. To enable this call, Federico appointed a panel of international reviewers with different expertise. Freda was the expert in basic and applied immunology. We worked together to empower the critical issue of careers for young scientists which is pivotal for the future of science. Discussing different aspects of science and life, we enjoyed our friendship.

## CONCLUSION

Surviving in the competitive world of research requires many strengths. Challenges shift for each generation but remain dominated by pressures on funding, especially to implement new technology, creating competition for limited resources. Perhaps gender equality has improved but social aspects are often a rather neglected area. International conferences used to be challenging and Freda had to watch the men go to the bar together and then out for dinner, leaving her to a lonely hotel. She solved this by contacting female participants beforehand and arranging dinners together. If Federico was at the conference, she was assured of social contact and a pleasant interactive dinner, with lots of catch up on CLL and immunology. We have been fortunate to be “friends in need” and we hope that this kind of support can be found by all in the research community.

## AUTHOR CONTRIBUTIONS

**Freda K. Stevenson**: Conceptualization; writing—original draft; writing—review & editing; validation. **Federico Caligaris Cappio:** Conceptualization; writing—original draft; writing—review & editing; visualization.

## CONFLICT OF INTEREST STATEMENT

The authors declare no conflict of interest.

## FUNDING

This research received no funding.

## References

[1] Caligaris‐Cappio F , Gobbi M , Bofill M , Janossy G . Infrequent normal B lymphocytes express features of B‐chronic lymphocytic leukemia. J Exp Med. 1982;155(2):623‐628. doi:10.1084/jem.155.2.623 6977012 10.1084/jem.155.2.623 PMC2186600

[2] Damle RN , Wasil T , Fais F , et al. Ig V gene mutation status and CD38 expression as novel prognostic indicators in chronic lymphocytic leukemia. Blood. 1999;94(6):1840‐1847. 10477712

[3] Hamblin TJ , Davis Z , Gardiner A , Oscier DG , Stevenson FK . Unmutated Ig VH genes are associated with a more aggressive form of chronic lymphocytic leukemia. Blood. 1999;94(6):1848‐1854. 10477713

[4] Chiorazzi N , Stevenson FK . Celebrating 20 years of IGHV mutation analysis in CLL. HemaSphere. 2020;4(1):e334. 10.1097/HS9.0000000000000334 32382709 PMC7000474

[5] Stevenson FK , Caligaris‐Cappio F . Chronic lymphocytic leukemia: revelations from the B‐cell receptor. Blood. 2004;103(12):4389‐4395. 10.1182/blood-2003-12-4312 14962897

[6] Apollonio B , Scielzo C , Bertilaccio MTS , et al. Targeting B‐cell anergy in chronic lymphocytic leukemia. Blood. 2013;121(19):3879‐3888. 10.1182/blood-2012-12-474718 23460614

